# AFM-IR of Electrohydrodynamically Printed PbS Quantum Dots: Quantifying Ligand Exchange at the Nanoscale

**DOI:** 10.1021/acs.nanolett.4c02631

**Published:** 2024-08-21

**Authors:** Lorenzo J. A. Ferraresi, Gökhan Kara, Nancy A. Burnham, Roman Furrer, Dmitry N. Dirin, Fabio La Mattina, Maksym V. Kovalenko, Michel Calame, Ivan Shorubalko

**Affiliations:** ‡ Transport at Nanoscale Interfaces Laboratory, Empa - Swiss Federal Laboratories for Materials Science and Technology, CH-8600 Dübendorf, Switzerland; § Institute of Inorganic Chemistry, Department of Chemistry and Applied Biosciences, ETH Zürich, CH-8093 Zürich, Switzerland; ∥ Departments of Physics and Biomedical Engineering, Worcester Polytechnic Institute, Worcester, Massachusetts 01609, United States; ⊥ Concrete and Asphalt Laboratory, Empa - Swiss Federal Laboratories for Materials Science and Technology, CH-8600 Dübendorf, Switzerland; # Laboratory for Thin Films and Photovoltaics, Empa - Swiss Federal Laboratories for Materials Science and Technology, CH-8600 Dübendorf, Switzerland; g Department of Physics and Swiss Nanoscience Institute, University of Basel, CH-4056 Basel, Switzerland

**Keywords:** colloidal quantum dots, ligand exchange, electrohydrodynamic printing, AFM-IR, lead sulfide, infrared spectroscopy

## Abstract

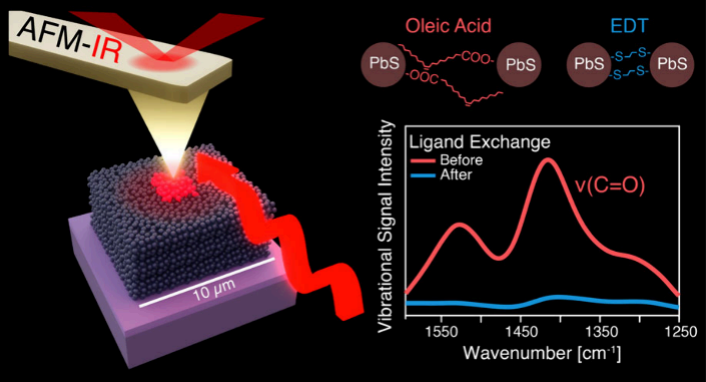

Colloidal quantum dots (cQDs), semiconductor materials with widely tunable properties, can be printed in submicrometer patterns through electrohydrodynamic printing, avoiding aggressive photolithography steps. Postprinting ligand exchange determines the final optoelectronic properties of the cQD structures. However, achieving a complete bulk exchange is challenging, and the conventional vibrational analysis lacks the required spatial resolution. Infrared nanospectroscopy enables quantitative analysis of vibrational signals and structural topography on the nanometer scale upon ligand substitution on lead sulfide cQDs. A solution of ethanedithiol led to rapid (∼60 s) exchange of ≤90% of the ligands, in structures up to ∼750 nm thick. Prolonged exposures (>1 h) caused the degradation of the microstructures, with a systematic removal of cQDs regulated by surface:bulk ratios and solvent interactions. This study establishes a method for the development of devices through a combination of tunable photoactive materials, additive manufacturing of microstructures, and their quantitative nanometer-scale analysis.

Colloidal quantum dots can be synthesized in and deposited from solutions into photosensitive films with semiconducting behavior. Their properties can be tailored through the choice of the core material, the core size, and the surface passivation.^1^ Together with the ease of fabrication, the tunable properties represent a great advantage for the fabrication of diverse devices, including photodetectors.^2^ Both material research and device engineering are needed for colloidal quantum dots (cQDs) to be integrated into a number of applications.

Lead sulfide cQDs have been extensively studied because of their size-tunable spectral sensitivity in the near- and short-wave infrared, as well as their accessible synthesis routes.^3−6^ As-synthesized cQDs are commonly dispersed in nonpolar solvents and coated by long, insulating ligands. These can be removed and substituted, with the choice of the final molecule taking into account surface passivation^7^ and energy-level tuning,^8^ together with interparticle distance,^9^ determining the transport properties of the material. There are two main methods for performing this molecular substitution: liquid-phase ligand exchange (LPLE) and solid-state ligand exchange (SSLE). They are defined on the basis of whether the exchange happens before or after the deposition of the cQD film.

In the SSLE treatment, the ligand solution is applied to a previously deposited film of cQDs. Its thickness is limited to a few nanometers with the aim of optimizing the exchange process, controlled by the penetration of the film by the ligand solution. Multiple layers are superposed to reach the required thickness.^6,10−12^ This laborious fabrication shifted attention to the LPLE, where the careful optimization of concentrations and solvents led to single-step depositions of conductive ligand-exchanged cQD films.^13^ Spin-coating, the deposition method of choice in both cases, results in a large amount of material waste, which is worsened by a layer-by-layer deposition.^14^ Further complexities arise once the active layer is structured and integrated into devices. Photolithography, widely used in fabrication processes, can easily harm the active layer by exposing it to aggressive gaseous or liquid environments.^15^

Microstructures of colloidal semiconductors can be additively manufactured through printable active inks, drastically improving the material economy. The absence of photolithographic structuring avoids damage to the active layer and overcomes limitations of device design. Devices were fabricated via inkjet printing using both SSLE^16^ and LPLE^17^ procedures. However, the best printing spatial resolution to date was obtained through electrohydrodynamic (EHD) printing. In contrast to inkjet printing, in which a pressure pulse is applied to the printing nozzle, EHD printing uses an ac electric field between the nozzle and the substrate.^18,19^ This electric field leads to the accumulation of cQDs in the meniscus at the nozzle opening. Eventually, an apex is formed (Taylor cone), pulling droplets considerably smaller than the nozzle opening. Via careful optimization of the properties of the ink, including its polarity, viscosity, and vapor pressure,^20^ precise micrometer^21^ and submicrometer^22^ pattern fabrication is possible.

When this optimization is achieved, EHD printing permits the downscaling of devices to few micrometers. Typically, nonpolar solvents are used for the best lateral printing resolution, so a postdeposition SSLE is required to obtain a conductive cQD film. Understanding the impact of ligand exchange processes on these printed microstructures is critical for imparting the needed functionalities. The key parameters to be monitored in this process are the total volume of structures and the residual presence of the native ligands. Chemical treatments can damage the network of inorganic quantum dots and organic ligands, causing losses of photoactive material with an associated volume variation. If cQDs are not lost, then the change in volume is proportional to changes in the ligand shell. The volume variations can be associated with the exchange of ligands through conventional IR spectroscopy, by monitoring the intensity of their unique vibrational signatures.^6,23−25^ Nevertheless, the lateral resolution of this technique is limited to micrometers by optical aberration. In contrast, the near-field AFM-IR technique provides for parallel analysis of both structural topography and chemical composition at the nanoscale.^26^

In this work, we develop a method that enables the quantitative study of ligand exchange processes at the nanoscale. The variations in both volume and chemical composition are considered simultaneously as measured through AFM-IR in tapping mode. Microstructures are fabricated by EHD printing of PbS cQDs with an excitonic peak at 1.6 μm. The surface of the cQDs is coated by oleic acid (OA) ligands, and they are dispersed in a nonpolar solvent (tetradecane) for the best results in terms of printing spatial resolution. Microstructures (nominal area of 10 μm × 10 μm) with different heights (125 ± 20, 470 ± 60, and 750 ± 90 nm) are printed on each of the samples. After printing, a one-step SSLE is applied to replace the insulating OA with ethanedithiol (EDT) and obtain a conductive material. This is chosen to optimize the lateral printing resolution with nonpolar solvents, while avoiding the fabrication disadvantages of a layer-by-layer approach. Different samples are exposed to the ligand solution for different time intervals (60 s, 1 h, and 12 h) to study the penetration of the ligand into the printed microstructures. Their high surface:volume ratio enhances the interaction with the ligand solution. 90% of the ligands can be exchanged in a surprisingly short time of 60 s, in both 125 and 750 nm structures. With an increase in treatment time, the removal of OA is not improved, while the ligand solution damages the active layer, removing quantum dots and damaging structures through cracks.

The ligand exchange process determines several properties of the final colloidal semiconductor film. It aims to improve charge-carrier transport between cQDs through the replacement of long, insulating molecules by short, conductive molecules that can still passivate the surface. This process will determine two main measurable effects: the volume contraction due to a reduced interparticle distance and the disappearance of vibrational features associated with the removed molecules. AFM-IR is ideally suited for this study as it allows the measurement of both effects simultaneously at the nanometer scale. This technique can overcome resolution limitations associated with conventional IR spectroscopy, because the wavelength exciting vibrational modes is on the same size scale as the analyzed microstructures (Figure 1a). The metal-coated AFM tip first enhances locally the incoming IR laser pulses, resulting in the excitation of vibrational modes in the OA ligands (Figure 1b). The consequent pulsed thermal expansion of the sample is proportional to the absorption coefficient and is detected mechanically by the tip following the same principle of conventional AFM topography scans. In particular, it can be measured at a fixed excitation wavelength while the tip is scanned across the sample, simultaneously generating topographic and chemical maps and identifying the IR-active regions (Figure 1c). Alternatively, the tip can be placed in a fixed position and the excitation wavelength can be scanned across the desired range to acquire vibrational spectra (Figure 1d).

**Figure 1:**
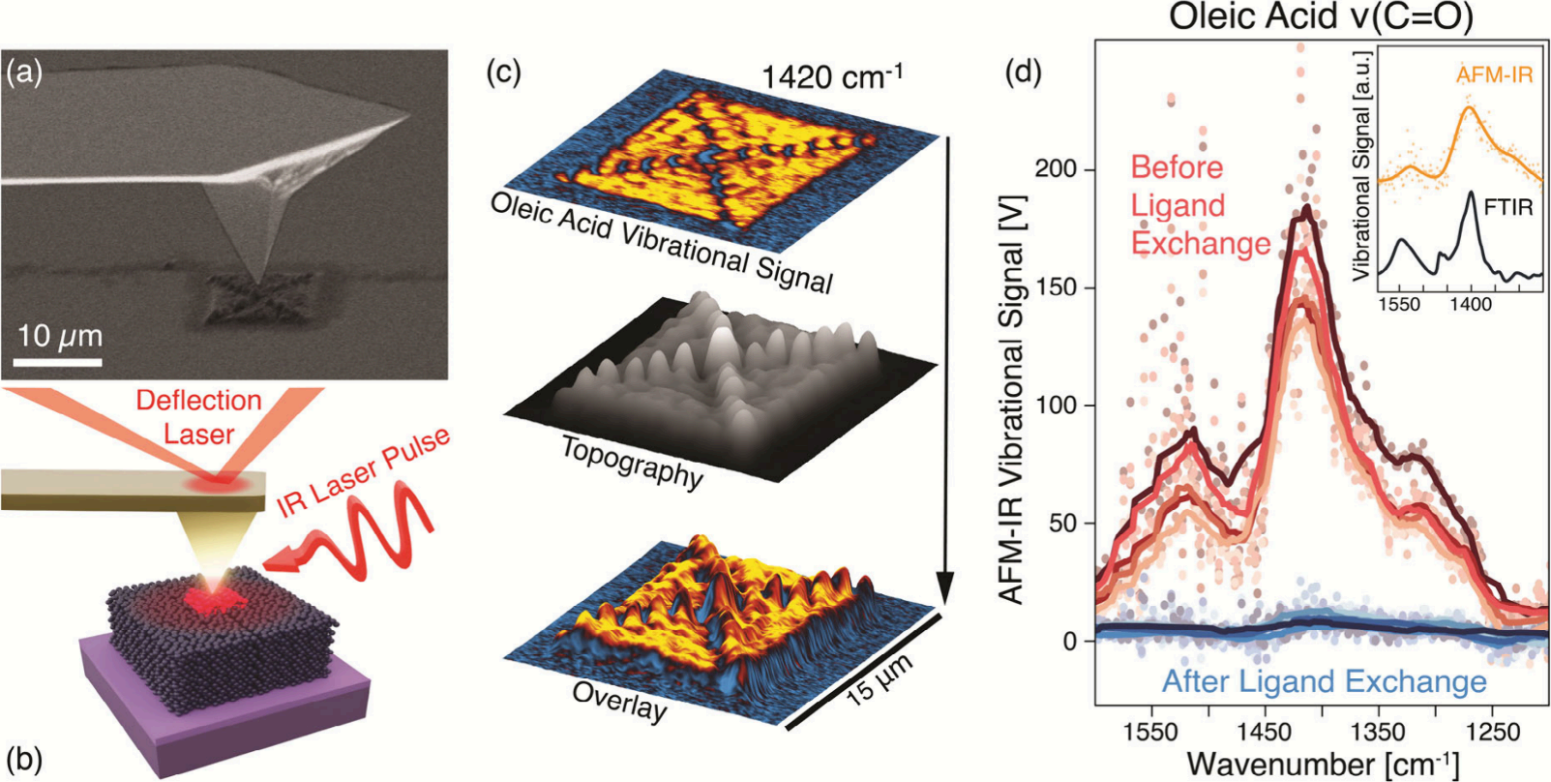
AFM-IR principle and detection of OA removal. (a) Scanning electron microscopy images of the tip used in this AFM-IR analysis and of the sample with the printed cQD microstructures (to scale; pictures were collected separately). (b) Schematic illustration of the AFM-IR technique (not to scale). The metallic tip enhances the incoming IR laser pulses (5–11 μm or 900–1900 cm^–1^). The IR light is absorbed through the vibrational modes of organic ligands, causing a pulsed thermal expansion detected mechanically by the AFM tip. Both conventional topography and vibrational topography are measured through the deflection laser. (c) Results from AFM-IR scans of structures, where chemical composition (top) and topography (middle) maps are combined to clearly locate targeted molecules on the surface (bottom). (d) AFM-IR spectra collected from a printed structure. The technique clearly detects both the as-printed signal from the C=O bond stretching (red) and the quenched signal once the ligand exchange treatment is performed (blue). Intensity values are reported in volts as provided by the tool. In the inset, the reference vibrational signal (black) is measured through conventional FTIR from a spin-coated film and can be recognized in the AFM-IR measurement of the same sample (yellow).

Carboxyl groups in OA are identified through the stretching mode of the C=O bond, with two main peaks at ∼1400 and ∼1550 cm^–1^ when the molecule is bound to the PbS cQDs surfaces.^23,24,27^ The results are consistent with conventional infrared spectroscopy, as both FTIR and AFM-IR spectra show the most intense vibrational peak at ∼1420 cm^–1^, with a smaller signal at ∼1500 cm^–1^ (inset of Figure 1d). Ligand exchange treatments severely quench the intensity because most of the oleic acid molecules are removed. Residual molecules can still be detected after the application of an EDT ligand exchange treatment (Figure 1d), and the degree of variation of the vibrational signal determines the quality of the applied treatment.

The relation between the concentration of the analyzed chemical species and the resulting vibrational signal intensity must be expressed. The resulting calibration curve can be used to quantify the molecules of oleic acid on the basis of the intensity detected through AFM-IR. In this work, different oleic acid concentrations correspond to different structure heights.

The AFM-IR setup may introduce undesired variations between measurements following laser power fluctuations and defocusing, resulting in distorted intensity values that do not correlate with the oleic acid concentration. To address this issue, a normalization procedure against a spin-coated sample is applied (see Methods in the Supporting Information for more details). This sample serves as a reference because of its uniformity and low roughness, resulting in small intrinsic signal variations across different specific regions of interaction with the AFM tip.

The AFM-IR scans show how the chosen printing path results in an overall uniform film while introducing a cross-like feature where excess cQDs are deposited (Figure 2a,b). The heights considered for this work neglect these features and refer to only the underlying plateaux. These features have an impact on the vibrational mapping, as they shade neighboring regions from the IR excitation, determining variations in the intensity of the vibrational signal related to the different excitation intensities, as reported in the literature^28^ (Figures 1c and 2b).

**Figure 2:**
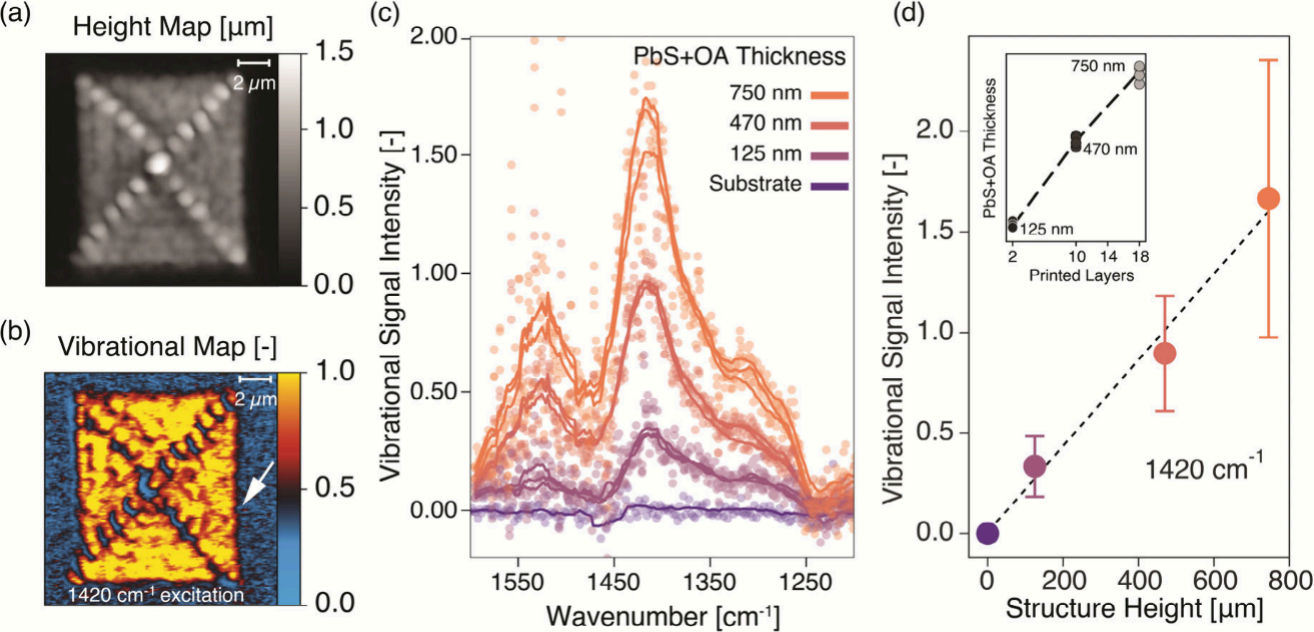
Topographic and vibrational information from EHD-printed PbS cQD structures before ligand exchange. (a) AFM-IR topography of a representative printed structure used to measure the average thickness of the active material. A thicker, cross-shaped feature can be noticed, unintentionally introduced due to the chosen printing procedure. (b) Normalized AFM-IR vibrational signal map, demonstrating the presence of resonating molecules only in the printed microstructure. The cross-shaped feature shades some regions from the excitation pulses, determining variations in signal intensity, which is noticeable in Figure 1c. The white arrow shows the direction of the laser pulses used for excitation, consistent with the shading. (c) Representative vibrational spectra collected from the uniformly excited regions of printed structures with different thicknesses, and normalized as described in Methods. Shaded regions, identified through the previously collected maps, were avoided. A spectrum was collected from the substrate (silicon oxide) to determine the intensity without the active material. The distortion visible in the substrate signal at ∼1450 cm^–1^ is related to the laser stage changing at 1449 cm^–1^. (d) Intensities at 1420 cm^–1^ as a function of the height of the structures, serving as calibration curve for further analysis. For each structure height, 26 spectra have been collected from different locations and their intensities averaged. A percentile-based error bar is provided (80%). A linear interpolation between average values is plotted as a black dashed line. The inset shows the measured heights obtained by overlaid printed layers on the four samples analyzed in this work. Each data point represents the height of a structure, with a total of 12 structures printed on four samples. Written values represent the average height of structures with the same number of superposed printed layers, with the black dashed line serving as guide to the eye.

The EHD printing of structures with 2, 10, and 18 cQD printing loops was repeated on each of the four samples. This layer superposition scheme corresponds to well-resolved average heights of 125 ± 20, 470 ± 60, and 750 ± 90 nm, respectively (inset of Figure 2d). The roughness of the microstructure surface, reported as the error in the height measurement, increases with the number of printing loops. Every layer interacts with the previous one due to the transitory presence of the solvent, leading to a larger roughness and a dispersion of height values.

The intensity of the vibrational signal at 1420 cm^–1^ as extracted from spectra is directly related to structure height, increasing as more functional groups are excited by the laser pulses and interact with the AFM tip (Figure 2c,d). Multiple spectra are collected from different regions of the structures undergoing uniform excitation conditions (26 for each substrate height), and then the extracted values are normalized by the reference signal. With no Gaussian distribution emerging from the collected data points, a percentile-based error has been chosen [80% (distributions shown in Figure S11)]. No saturation of the vibrational signal intensity is observed as the height of the structure increases (Figure 2d), suggesting that the penetration depth of the excitation laser is sufficient to probe the entire volume of the analyzed structures. The factors determining the observed dependence may include the relation between absorbed light and temperature gain in films with different thicknesses, and the distribution of heat in the cQD film due to the presence of different interfaces. With constant settings, a constant setup, and a constant sample structure, calibration curves allow the residual amount of oleic acid remaining after the different ligand exchange procedures to be measured.

The overall quality of the ligand exchange process is studied here considering both volume losses and oleic acid vibrational signal variations. The structures are exposed to the same ligand solution for increasing amounts of time (60 s, 1 h, and 12 h), to evaluate if a short interaction time is a limit for a complete exchange and if long exposures improve results or rather introduce damage. Three structure heights are investigated (125 ± 20, 470 ± 60, and 750 ± 90 nm) to assess the role of the initial volume in the interaction with the ligand solution.

Volume variations in the microstructures are expected, following the reduction of the PbS cQD interparticle distance under ligand exchange from OA to EDT. These variations can be modeled through the contracting random loose packing model, developed for printable inks of metallic nanoparticles.^29^ Different interparticle distances have been reported for PbS, depending on whether ligand shells partially merge^30^ (from 2.6 nm with OA to 1.2 nm with EDT) or remain fully isolated^31^ (from 4 to 1.6 nm).^32^ Considering the case of partially merged ligand shells and PbS cQDs with a core diameter of 6 nm, a complete ligand exchange would correspond to a 20% volume loss. The volume loss is directly translated into height loss, considering the in-plane shrinkage as negligible due to the adhesion with the substrate. The extended treatment, following the models by Sattler et al.,^29^ can be found in the Supporting Information (paragraph S10).

Topography scans are used to measure the height variations and to check for structural damage. The shortest ligand treatment of 60 s is the most controllable and homogeneous, resulting in height variations between 26% and 28% for structures between 125 and 750 nm (Figure 3a). This is consistent with the replacement of ligands, plus an initial degree of cQD loss, pushing the overall volume loss only slightly beyond the modeled value.

**Figure 3:**
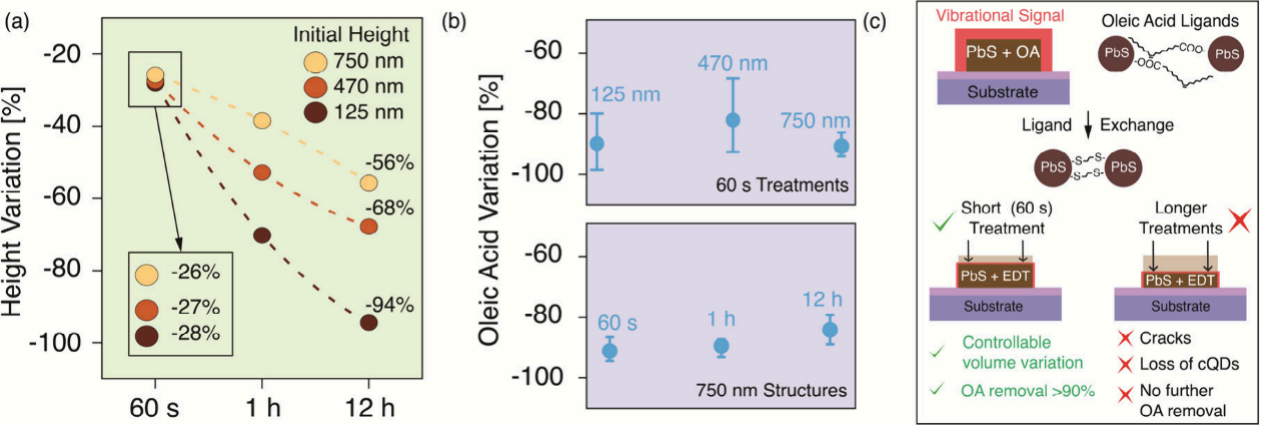
Structure height and oleic acid vibrational signal variations with different ligand solution exposure times. (a) Height variation in structures with different initial heights. The expected level of −20% volume loss is calculated on the basis of the random loose packing model. The shortest treatment introduces similar volume losses into different structures, suggesting a controllable process with ligand substitution prevailing over structural degradation. Longer treatments introduce excessive losses associated with active layer damage, with a clearer dependence on the surface:volume ratio of structures. Numbers are obtained through the histogram of height values in AFM topographic maps. (b) Oleic acid vibrational signal variation upon ligand exchange. Short treatments of 60 s result in a >90% loss of oleic acid for both 125 and 750 nm structures (top). The ligand exchange of 750 nm structures is not improved beyond 90% with an increase in treatment time (bottom). Between six and nine spectra from different locations are considered for each variation, with the graph reporting average values and error bars including the whole set of data points. (c) Effects of ligand exchanges of different durations on the as-printed structures (not to scale). The shorter treatment of 60 s results in the substitution of ligands, associated with the removal of the vibrational signal from oleic acid molecules and the reduction of the interparticle distance, causing the negative height variations in structures. Treatments with excessive durations result in degradation of the active structures, with no further removal of the oleic acid residual signal.

Excessive volume losses are measured when the cQD structures are exposed to the ligand solution for longer periods of time. The surface:volume ratio of the microstructures in this case determines the clearer differences in the interaction with the ligand solution. When left overnight in the EDT solution, the 125 nm structures are almost entirely removed (−94%), while 470 and 750 nm structures lose 68% and 56% of their volumes, respectively. These volume losses and their dependence on the surface:volume ratio are consistent with the substantial removal of the active material from the microstructures, which is detrimental to the final device performance. The same conditions also result in the formation of cracks (Figure S3), possibly creating short circuits if such printed structures are then integrated into vertical devices. No damage is instead visible in any microstructure when a 60 s ligand treatment is applied. The information on volume loss must be coupled with the measurement of the OA vibrational signal variation to understand in more detail the ligand exchange process and determine its efficiency.

The variation in the vibrational signal is analyzed as a function of the height of microstructures in the case of a 60 s treatment (Figure 3b). The signal loss in this case is >90% for both 125 and 750 nm structures. The structural damage and active material loss induced by treatments of >60 s may result in larger variations between samples and between different areas of the same structure. On the basis of our measurements, increasing the treatment duration does not improve the ligand exchange in thicker structures (750 nm), as the measured oleic acid signal does not change significantly from that observed after 60 s.

Two processes with different time constants can be considered to explain the observed behavior: the exchange of the ligand shell around cQD cores, completed within the first minute, and the undesired removal of cQD cores with their ligand shell from the printed structures, developing over hours. On the basis of the random loose packing model, the change in the ligand shells from oleic acid to EDT accounts for 18% of height loss when 90% of the oleic acid is removed. A minor degree of cQD loss (8–10% of the initial volume) is then obtained in 60 s when signal variation confirms that the ligand exchange process has already reached saturation. Longer treatments further damage the structures while not improving the removal of oleic acid. A sigmoidal dependence between film properties and ligand solution concentration was demonstrated in previous reports.^33^ The 2% volume concentration of EDT ligands applied in this work determines the time scale of the ligand exchange process, which appears to be faster than the minimum treatment duration of 60 s. The leftover signal from oleic acid molecules might be related to a layer of cQDs at the substrate interface, where oleic acid ligands can bind to the oxide substrate.

Overnight exposure to ACN only was performed as a control experiment to decouple the effect of ligands and solvent (Figure S8 and paragraph S9). Although some reports showed that ACN does not interact with the ligands,^34^ this solvent has been chosen previously to optimize SSLE processes because of its interactions with the cQD films. In particular, cQDs can rearrange themselves in the layer during postdeposition ACN treatments, leading to self-curing of cracks and defects.^35^ The longer exposure time applied in this work instead drives solvent degradation of the photoactive cQD microstructures.

In conclusion, AFM-IR allowed us to quantitatively assess the ligand exchange efficiency in the challenging case of EHD-printed microstructures of PbS cQDs beyond the capabilities of conventional FTIR measurements. For this, 10 μm × 10 μm structures with varying volumes were analyzed by simultaneously investigating their topography and vibrational signatures. With the chosen ligand concentration and structure dimension, the preferred treatment is the shortest. Only 60 s is needed to reach saturation and remove 90% of the oleic acid in the 125 and 750 nm structures. This is obtained with minimal active material losses, quantified as 8–10% of the initial volume and no structural damage. Longer exposures to the ligand solution incur deviations from the modeled volume loss. The undesired removal of the photoactive cQDs takes over, introducing structural damage without improving the substitution of surface molecules. Through the combination of microscale additive manufacturing and nanoscale quantitative analysis, this work provides important knowledge for the development of solution-processed devices based on conductive colloidal semiconductors.

## Supporting Information Available

Methods, absorbance of cQDs, AFM-IR data collection procedure, AFM topography scans, normalized AFM-IR spectra, AFM-IR spectrum of the substrate, reference signal variations, control experiment under pure ACN exposure, random loose packing model, distributions of vibrational signal intensity values, and a histogram of the height measurements (PDF)
