# Supplementary material for: AFM-IR of Electrohydrodynamically Printed PbS Quantum Dots: Quantifying Ligand Exchange at the Nanoscale
Source: Nano Lett. 2024 Aug 21;24(35):10908–14. doi: 10.1021/acs.nanolett.4c02631 (PMC11378332; doi:10.1021/acs.nanolett.4c02631)
Supplement: Supplementary file 1 — nl4c02631_si_001.pdf [file nl4c02631_si_001.pdf]

Supporting Information

AFM-IR of EHD-Printed PbS Quantum Dots: Quantifying Ligand Exchange at the Nanoscale

Lorenzo J. A. Ferraresi[a,b,c], Gökhan Kara[a,b], Nancy A. Burnham[e,f], Roman Furrer[b], Dmitry N. Dirin[c,g], Fabio La Mattina[b], Maksym V. Kovalenko[c,g], Michel Calame[b,d], Ivan Shorubalko*[b]

(a) These authors contributed equally

(b) Transport at Nanoscale Interfaces Laboratory, Empa – Swiss Federal Laboratories for Materials Science and Technology, CH-8600 Dübendorf, Switzerland

(c) Department of Chemistry and Applied Biosciences, ETH – Swiss Federal Institute of Technology Zurich, CH-8093 Zurich, Switzerland

(d) Department of Physics and Swiss Nanoscience Institute, University of Basel, CH-4056 Basel, Switzerland

(e) Departments of Physics and Biomedical Engineering, Worcester Polytechnic Institute, Worcester, Massachusetts

(f) Concrete and Asphalt Laboratory, Empa, Swiss Federal Laboratories for Materials Science and Technology, Dübendorf, Switzerland

(g) Laboratory for Thin Films and Photovoltaics, Empa – Swiss Federal Laboratories for Materials Science and Technology, CH-8600 Dübendorf, Switzerland

* *ivan.shorubalko@empa.ch*

Methods

PbS cQD synthesis

PbS QDs were synthesized with slight adaptions to the method described by Hines et al.[1] as described previously.[2] Spectra of the PbS cQDs dispersed in tetrachloroethylene were acquired by UV-Vis spectroscopy (Jasco V-670) (SI Figure S1), highlighting an excitonic peak at ~0.77 eV.

Spin coating of reference sample

The as-synthesized PbS cQDs were redispersed in octane (240 mg/ml) and filtered with a 0.1 µm PTFE syringe filter. The drop was placed on the substrate while spinning it at 1500 rpm for 45 s. The high cQD concentration is chosen to obtain a thick layer of particles (~260 nm) in a single step.

Ligand Exchange Procedure

The native ligands were exchanged by a solid-state ligand exchange treatment with 2% vol. ethane-

1,2-dithiol (EDT) in acetonitrile. For the 60 s treatment, the sample was placed on a spinner and fully covered by a drop of EDT in acetonitrile (~ 150 µL). After 60 s, the sample was spin-dried at 2500 rpm for 45 s. The sample was rinsed with 5 drops of pure acetonitrile while spinning at 2500 rpm for 45 s. For the longer treatments, the samples were soaked in EDT in acetonitrile for 1 h or overnight. The EDT-treated samples were then dipped in pure acetonitrile and blow-dried with N_2. Subsequently, all the samples were placed on a spinner and rinsed with five drops of pure acetonitrile while spinning at 2500 rpm for 45 s. The overnight treatment was also performed in pure acetonitrile as a control experiment.

AFM Structure Height Measurements

The Anasys nanoIR2 platform was used. Each of the printed structures was scanned in tapping mode with a rate line within 0.3-1 Hz and resolution between 13-17 pixels per micrometre. The cantilever of choice is the silicon gold-coated PR-EX-TnIR-A10 (Anasys), with spring constant of 1-7 Nm^{-1}, resonant frequency around 75 kHz and tip radius of ~30 nm. The measured heights were obtained by considering the histogram of height values for each structure (SI Figure S12), with the first two main peaks representing the substrate and the main plateau of structures. The difference between these two peaks is reported here as the structure's height. In this way, the cross-like feature is neglected.

EHD printing

The as-synthesized PbS cQDs were redispersed in n-tetradecane at a concentration of 40 mg/ml and filtered with a 0.1 µm PTFE syringe filter. They were then deposited using electro-hydrodynamic printing (EHD) with a commercial system (Scrona NanoDrip™ R&D Print System and a Gen11p51 print head).

The Scrona print head is a MEMS print head manufactured from silicon wafers. In comparison to single-nozzle capillary-like print heads used in many other studies voltage is not only applied between the substrate and the nozzle but there is an additional electrode embedded in the print head itself that is situated between at a small distance from the nozzle, facing the substrate chuck. The voltage V_{nozzle} applied to this electrode ("nozzle Voltage") will cause droplet ejection if the difference to the voltage applied to the ink at the print head ("Print head Voltage") is sufficiently large. As the droplets are ejected and pass across the extraction electrode they

will be guided straight downwards given the uniform electric field that is formed between the approximately 1 mm wide extraction electrode and the chuck which is driven at V_{chuck}. In this way, even at the used printing distance of 500 µm to the substrate accurate placement is secured.

The SiOx sample (525 µm thick silicon wafer with 280 nm Oxide) was glued and grounded using silver paste onto an ITO glass substrate, which was grounded to the printing system. The starting off-state voltage levels which are applied to precondition the ink meniscus are the following: V_{chuck} of 500 V, V_{nozzle} of 150 V, 20 V at print head (V_{PH}), 3 ms pulse duration, with nozzle pulse off duration set to 0 ms. In this way, the effective applied voltages are switched each 3 ms from V_{chuck} of 500 V, V_{nozzle} of 300 V, V_{PH} of -250 V to V_{chuck} of -500 V, V_{nozzle} of -300 V, V_{PH} of 250 V. The distance between print head and ITO glass next to sample was set to 500 µm. The system employs two complementary objectives: a bottom optic positioned beneath the substrate, enabling in-situ process monitoring exclusively with transparent substrates, and a top optic for post-process print examination. As our substrate was non-transparent, we initially calibrated the printing process on transparent ITO material before transitioning to the non-transparent substrate. Printing was initialized by increasing nozzle and PH voltage (to V_{nozzle} of ~300 V, V_{PH} of ~250 V) on ITO glass until a spot size of ~2 µm was achieved (observed with bottom microscope). When a stable printing pattern could be achieved, the print head was moved to the substrate with same relative distance to its surface (checking the orientation with top microscope), and printing started with +100 V higher V_{chuck} voltage (the oxide layer allows higher chuck voltages applied, resulting in better printing quality).

AFM-IR Vibrational Analysis

Vibrational signals were collected on the same platform under ambient conditions. The vibrational spectra were collected between 912 and 1900 cm^{-1} with a resolution of 2 cm^{-1} and 128 co-averages per point. The QCL emission profile was collected and considered as background to be subtracted from the spectra. The laser pulse rate is set at 1300 kHz with ~80 ns pulse width. The main parameters used to obtain the desired vibrational signal quality are the IR tuneable laser power (set at 5 % of available range) and piezo drive strength (set at 20 % of available range). The piezo drive strength controls the tapping oscillation amplitude, with larger values leading to better signal to noise ratio. These two parameters are balanced to avoid either thermal (laser- induced) or mechanical (tip-induced) damage to the imaged structures. The centre frequency at ~360 kHz and frequency range of 50 kHz were in the expected range for tapping-compatible AFM-IR probes.

To perform a quantitative study of the vibrational data an analysis procedure was purposefully developed in this work. The complete procedure of data collection and analysis is presented in Figure S2 and summarised here. The vibrational spectrum for the analysed material is obtained from conventional FTIR measurements of a reference spin-coated sample with a PbS-OA cQD layer thickness of ~260 nm. This allows the peak at 1420 cm^{-1} to be identified as the most intense vibrational feature. Once samples are brought to the AFM-IR platform, the topography allows identifying the location of the cQD structures. The quantum cascade laser is set to excite the vibrational feature at 1420 cm^{-1}, and IR maps are collected by scanning the AFM tip. Once the vibrational signal intensity is optimised on the maps, the AFM tip is placed in a fixed position, and the excitation wavelength of quantum cascade laser is varied to cover the desired range and collect vibrational spectra.

The reference sample provides vibrational signals with minimal intensity variations across different measurement sessions due to the uniform distribution of OA capped cQDs, avoiding large variations due to tip positioning, and to the low roughness, avoiding shading effects. Spectra are collected from the reference before and after every measurement on printed structures. The reference vibrational signal will then highlight

variations resulting from unstable laser power, from progressive contamination of the AFM tip, or from defocusing of the excitation beam during measurements. Representative variations in the reference signal are reported (SI Figures S6-7).

As both the vibrational and topographic nanoscale data collection happens through the AFM tip, all measurements are extremely sensitive to local properties, which may not be representative of the whole structure. Multiple measurements are thus collected from each microstructure in order to account for variations in vibrational signal due to different amounts of OA molecules or to shading of the excitation source. The AFM-IR spectra are represented including the collected data points as scatter plot, and a fitting curve obtained by filtering out spikes (excluding data points with variation exceeding 30%) and then applying a linear Savitzky-Golay filter with a window of 11. Intensity values of the 1420 cm^{-1} peaks are extracted from the fitted curve. The points extracted from every measurement are then averaged, and the percentile-based error (80%) is reported. Average values are used to calculate variations due to ligand exchange processes.

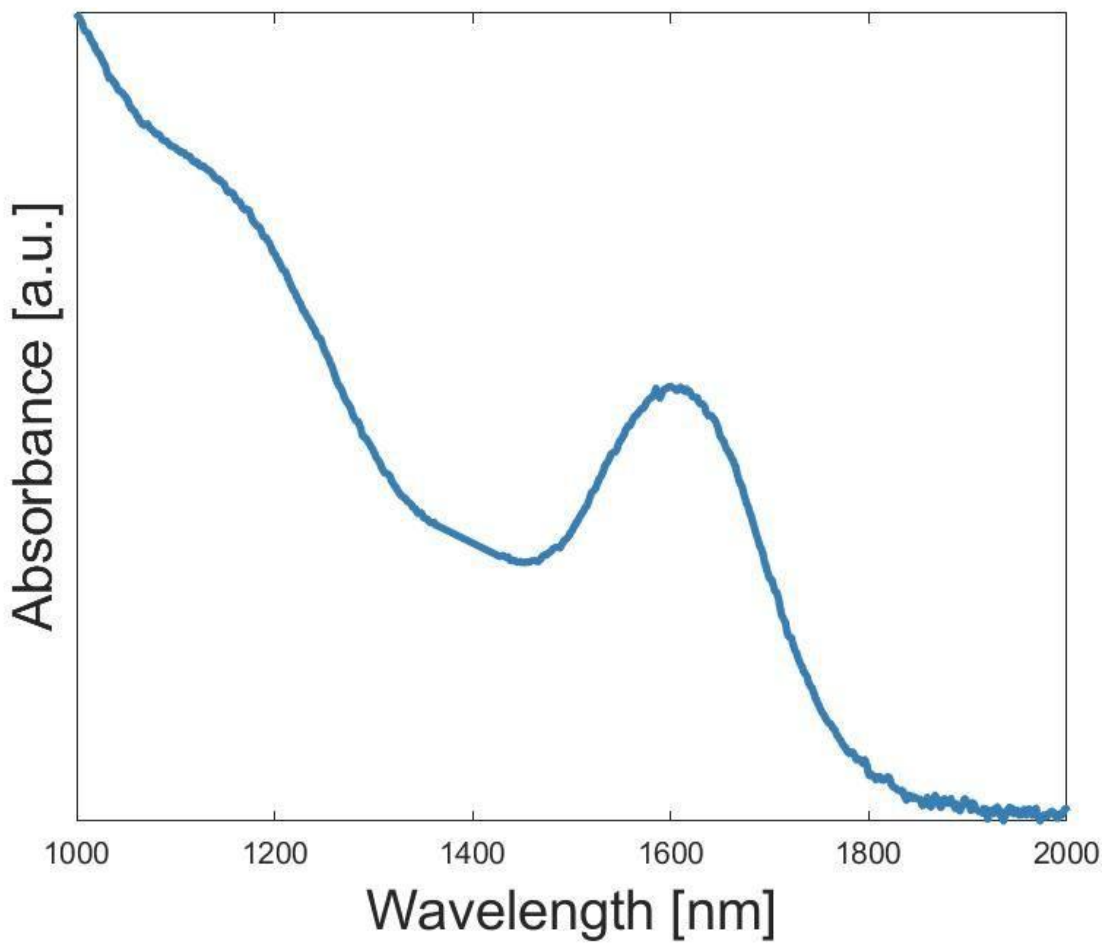

Figure S1: Absorbance spectrum of the PbS cQD solution used in this work, showing their well-defined excitonic feature around 1605 nm, corresponding to an energy of ~0.77 eV.

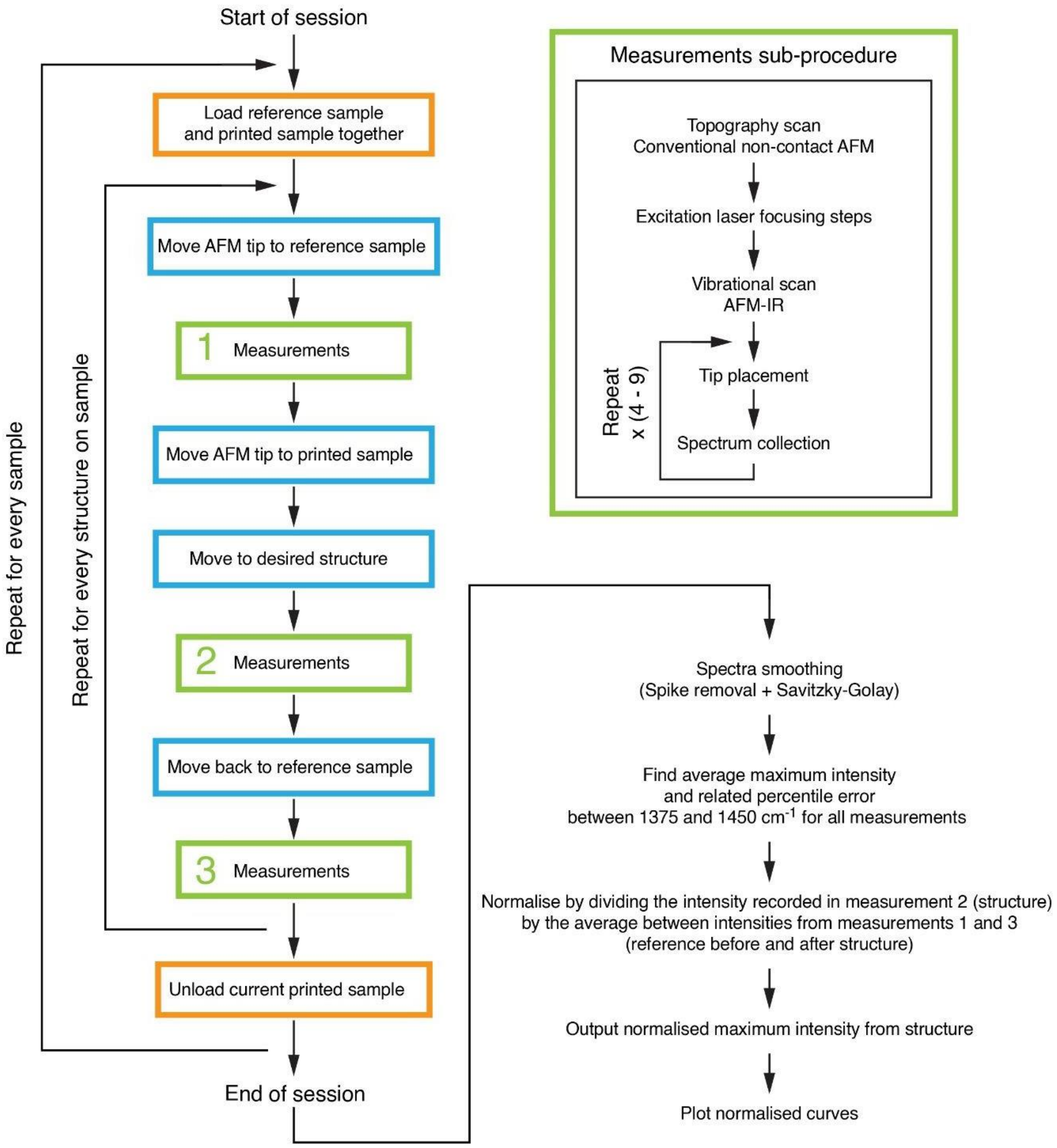

Scheme S2: Complete procedure for AFM-IR data collection and data treatment. This procedure follows the identification of the vibrational signal at 1420 cm^{-1} as the most intense through conventional FTIR. Data collection steps are colour coded as follows: load and unload (orange), movements of AFM tip (blue), measurements (green). Top right inset shows the sub-procedure of each measurement step. Vibrational scans are acquired with a fixed excitation wavelength and moving AFM tip, while vibrational spectra are acquired with a fixed AFM tip position and variable excitation wavelength.

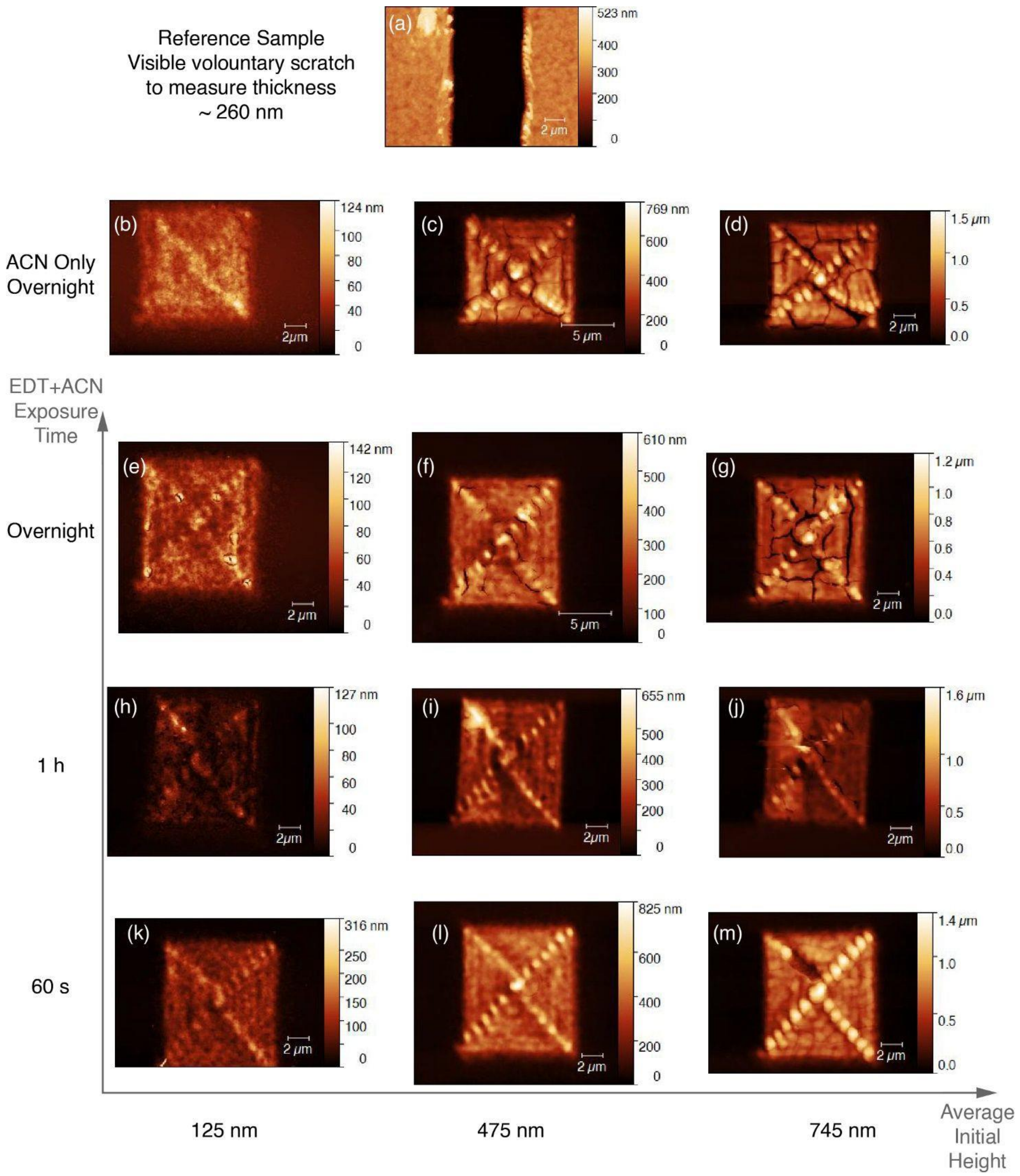

Figure S3: AFM topography scans of every analysed structure after ligand exchange, including reference sample. Every row corresponds to a sample, and in printed sample increasing printed structure heights are found from left to right. Refer to Figure 3(a) for the height variation introduced by the ligand exchange. (a) Spin-coated reference sample with scratch voluntarily introduced to expose the substrate and measure the thickness of the ligand-exchanged cQD film. (b)-(d) Sample exposed to ACN only. (e)-(g) Sample exposed to EDT in ACN overnight. (h)-(j) Sample exposed to EDT in ACN for 1 hour. (k)-(m) Sample exposed to EDT in ACN for 60 second. The large defect in (m) on the top-left arm of the cross-like structure has been introduced during measurements, and is caused by mistakes in handling of samples.

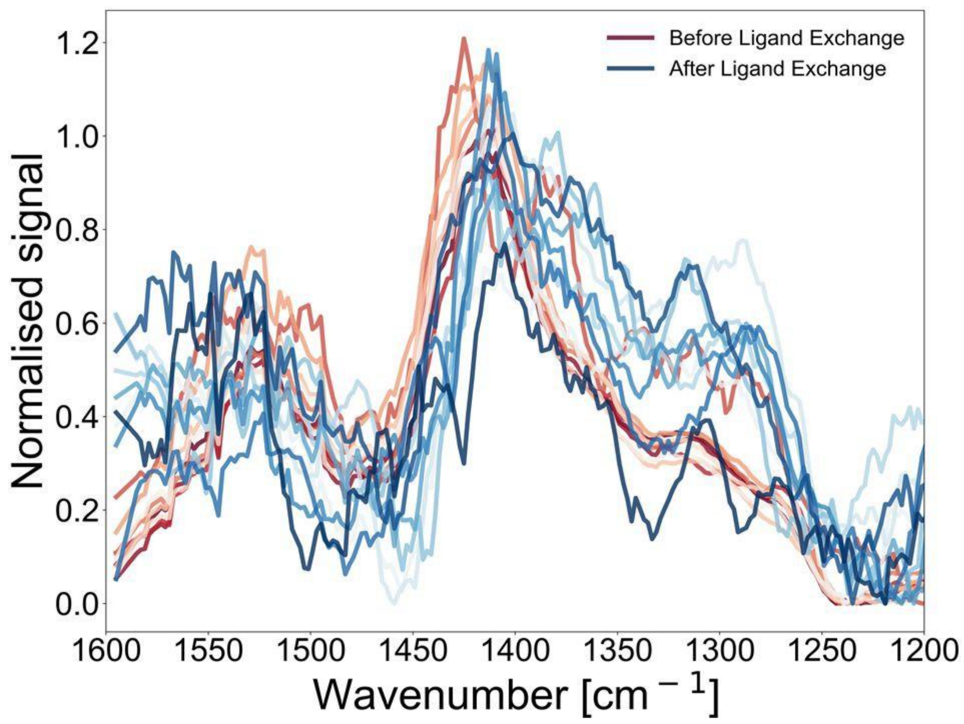

Figure S4: Comparison between normalised AFM-IR spectra before (red) and after (blue) ligand exchange. The measured structure has average height of 745 nm, on the sample exposed to EDT in ACN for 60 seconds. Spectra have the same profile, but the signal to noise ratio is lower after ligand exchange as ~90% of the signal is removed. The same spectra normalised to the reference sample only are visible in Figure 1(e).

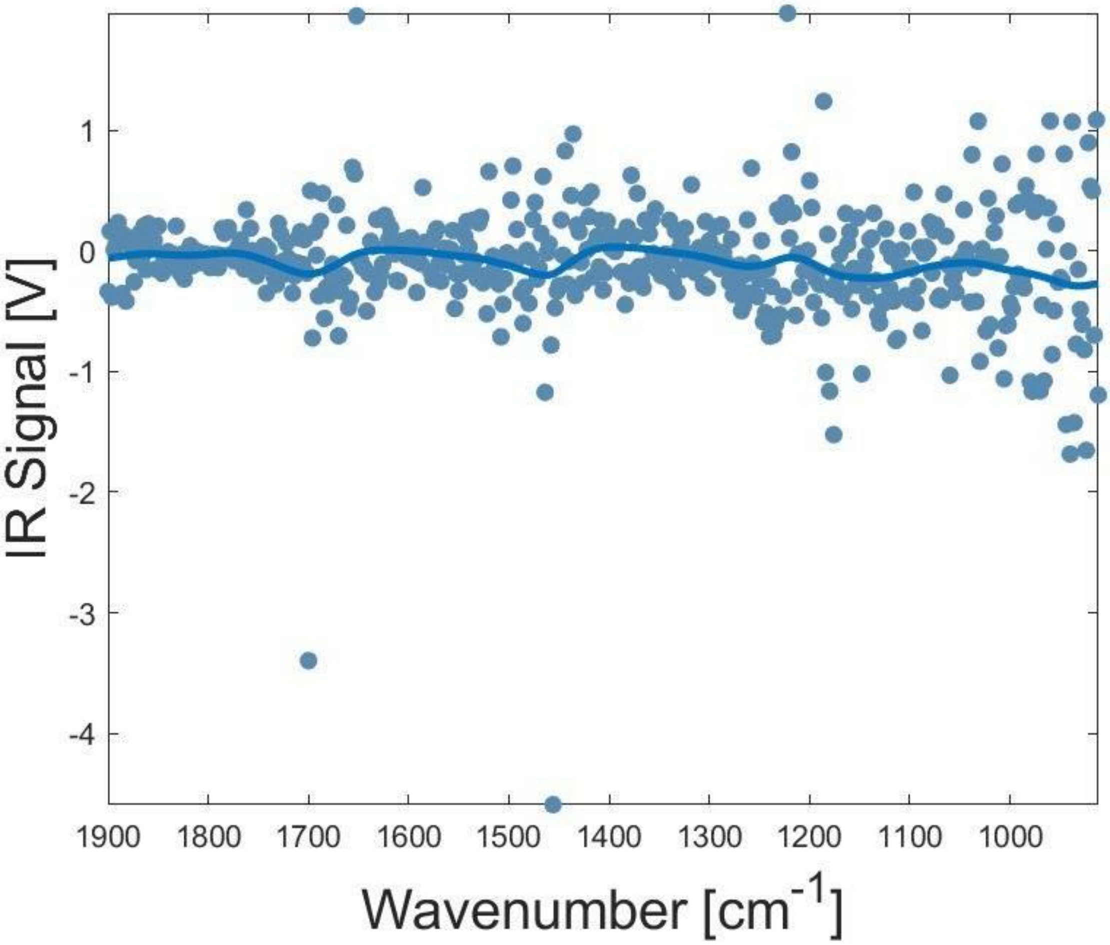

Figure S5: AFM-IR vibrational spectra measured from the substrate of PbS cQDs structures (silicon dioxide). No signal can be highlighted, as values of intensity remain close to zero. The oscillations are due to the excitation source, as four different lasers are needed to cover the full wavenumber range, and the transitions between them are visible in the detected signal. Intervals are the following (values expressed in cm^{-1}): 902-1180, 1180.1-1449, 1449.1-1700, 1700.1-1958.

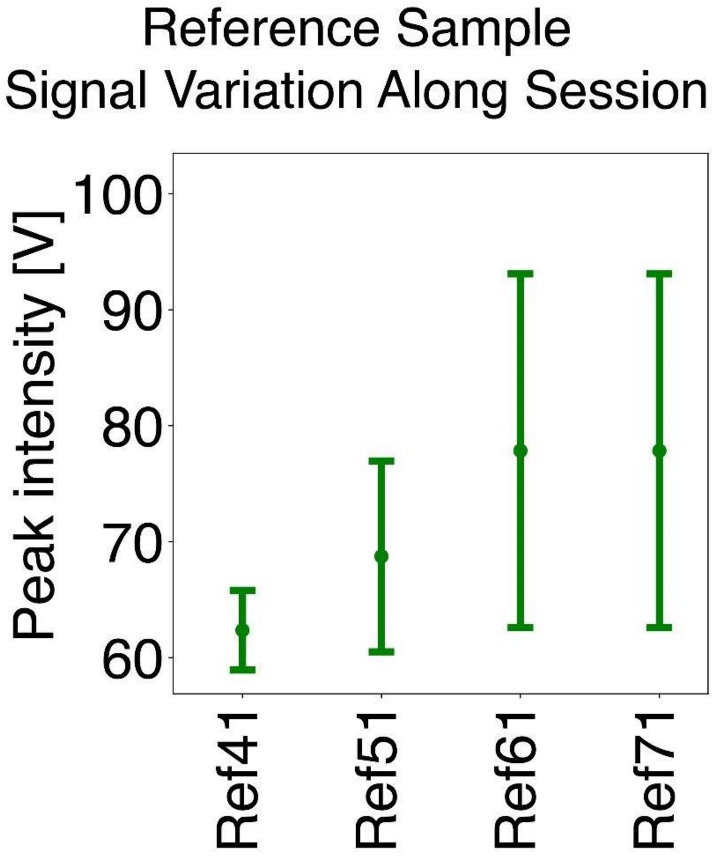

Figure S6: The vibrational signal from the reference sample used for normalisation along the measurement session of as-printed structures. The values are reported in volts as provided by AFM-IR setup.

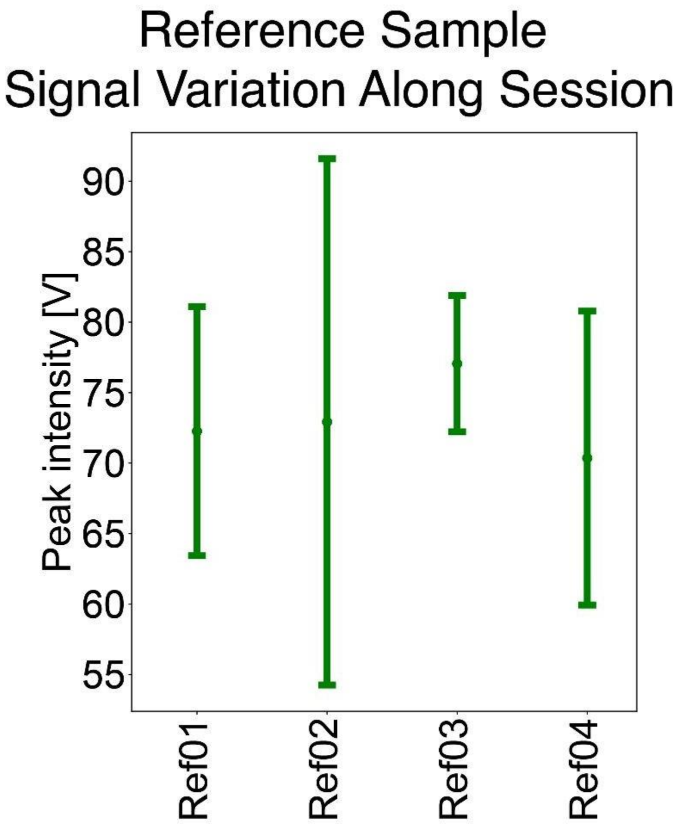

Figure S7: The vibrational signal from the reference sample used for normalisation along the measurement session of ligand-exchanged structures. The values are reported in volts as provided by AFM-IR setup.

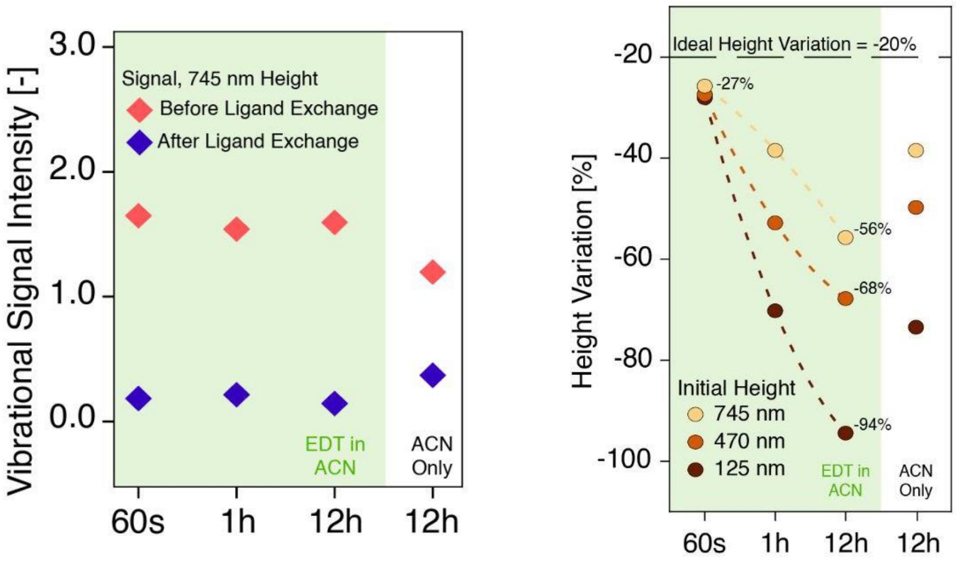

Figure S8: Control experiment with ACN only exposure. Variation of vibrational signal intensity for 745-nm-high microstructures upon the application of different ligand exchange procedures (left). Average values of intensities are reported. ACN treatment results in a 68% drop in oleic acid vibrational signal intensity. Variation of structure height for every structure height and ligand treatment (right). The ACN exposure results in lower height loss for structures, but still shows a dependence on their surface-to-volume ratio and exceeds the expected variation for the given percentage of oleic acid removal.

S9: Control experiment with ACN exposure

Overnight exposure to ACN had the specific goal of determining whether the volume contraction observed in the structures could be attributed to the replacement of OA ligands by EDT, or was just determined by the solvent washing away OA ligands, with no new capping molecules able to passivate the cQDs. The interaction in this case is milder, but results are still consistent with the loss of active material from the microstructures. A dependence on surface-to-volume ratio is still present, and the levels of contraction exceed the ones expected for the complete removal of ligand shells (~50% volume loss).

When considering the control sample exposed to ACN only, the removal of the vibrational signal drops to 68%. This percentage of OA removal, with no substitution by shorter molecules, would be consistent with a height loss of 34%, while the measured one is 40%, indicating partial loss of the active material. Excessive volume loss and cracking can also be caused by overnight exposure to ACN, suggesting that it damages the active layer without removing oleic acid.

S10: Random Loose Packing Model

The model applied in this work to estimate the effect of ligand exchange process on printed feature topography has been developed for metallic nanoparticles with organic ligands (gold NPs with alkyl thiol chains of different lengths).[3]

First, the shell thickness is a function of the number of carbon atoms in ligands, and of the nanoparticle radius. Considering oleic acid (18 carbon atoms) and a core radius of 3 nm, the model suggests a shell thickness of 1.5 nm. Literature reports show an inter-particle distance of 2.6±0.4 for PbS-OA through x-ray scattering

measurements.[4] This means the ligand shells are partially inter-penetrated, bringing cQDs slightly closer than the distance established by two isolated shells.

The EDT molecule is made by only two carbon atoms and two thiol groups. We main consider two options:

- The EDT molecule cross-links the cQDs: the expected inter-particle distance is below 1 nm
- The EDT molecule binds to a cQD and to the EDT molecule from another cQD: the expected inter-particle distance is ~1 nm

The reported inter-particle distance of 1.2±0.1 nm for PbS-EDT is consistent with the second scenario, where on average cQDs are actually not cross-linked, but a bond between sulphurs is created to obtain completely isolated shells. In fact, the ethane-monothiol can bring cQDs closer at 0.5+-0.2 nm.[2] Here, the shells appear to be partially inter-penetrated, as for oleic acid, thanks to the absence of a second SH group available to form a bond between thiols.

For this work, the sintering process studied by Sattler et al.[3] translates as the process of ligand exchange towards smaller molecules. The beta factor is the transformation parameter representing the progress of sintering. In this work, the largest possible reduction in inter-particle distance from OA to EDT (from 2.6 to 1.2 nm) can be visualised as a 50% sintered sample, so beta equals 0.5. The initial ratio between the shell thickness and the core radius ratio is also ~0.5. In this case, the model points to a height variation of -20% upon ligand exchange. This value is used here as ideal height variation value, representing a complete ligand exchange process with absence of active material loss.

For the control experiment where ACN solvent was applied in absence of EDT ligands, this reference level does not hold anymore: no ligands are supposed to be removed, and in case they are, no ligands are available to replace them. In case of complete ligand removal (corresponding to fully sintered metallic nanoparticles, or beta=1), the modelled volume loss is 50%.

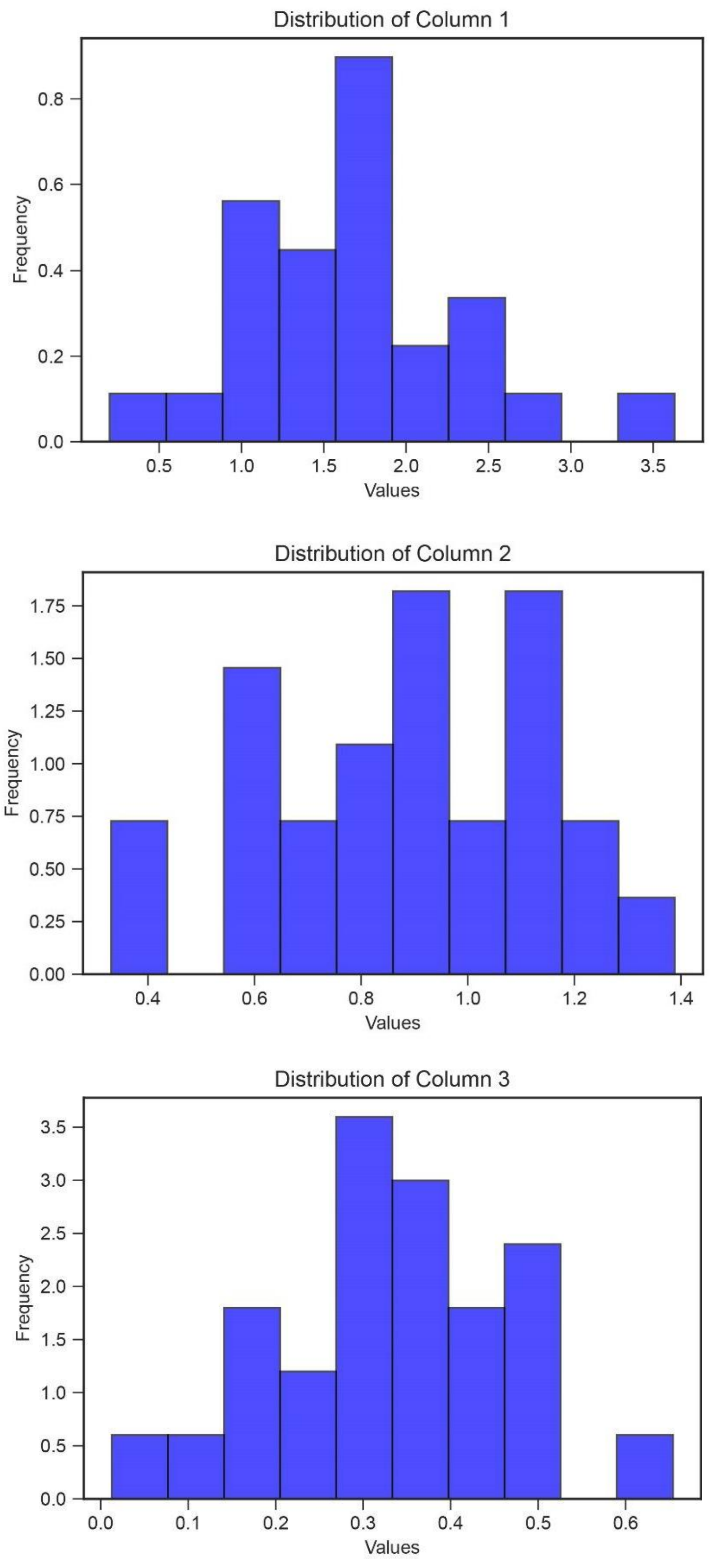

Figure S11: Distributions of the vibrational signal intensities for structures with different heights (745 nm top, 470 nm middle, 3125 nm bottom) before ligand exchange. Each distribution includes the 26 values of vibrational signal intensity at 1420 cm^{-1} obtained from AFM-IR spectra measured in different points on the printed structures.

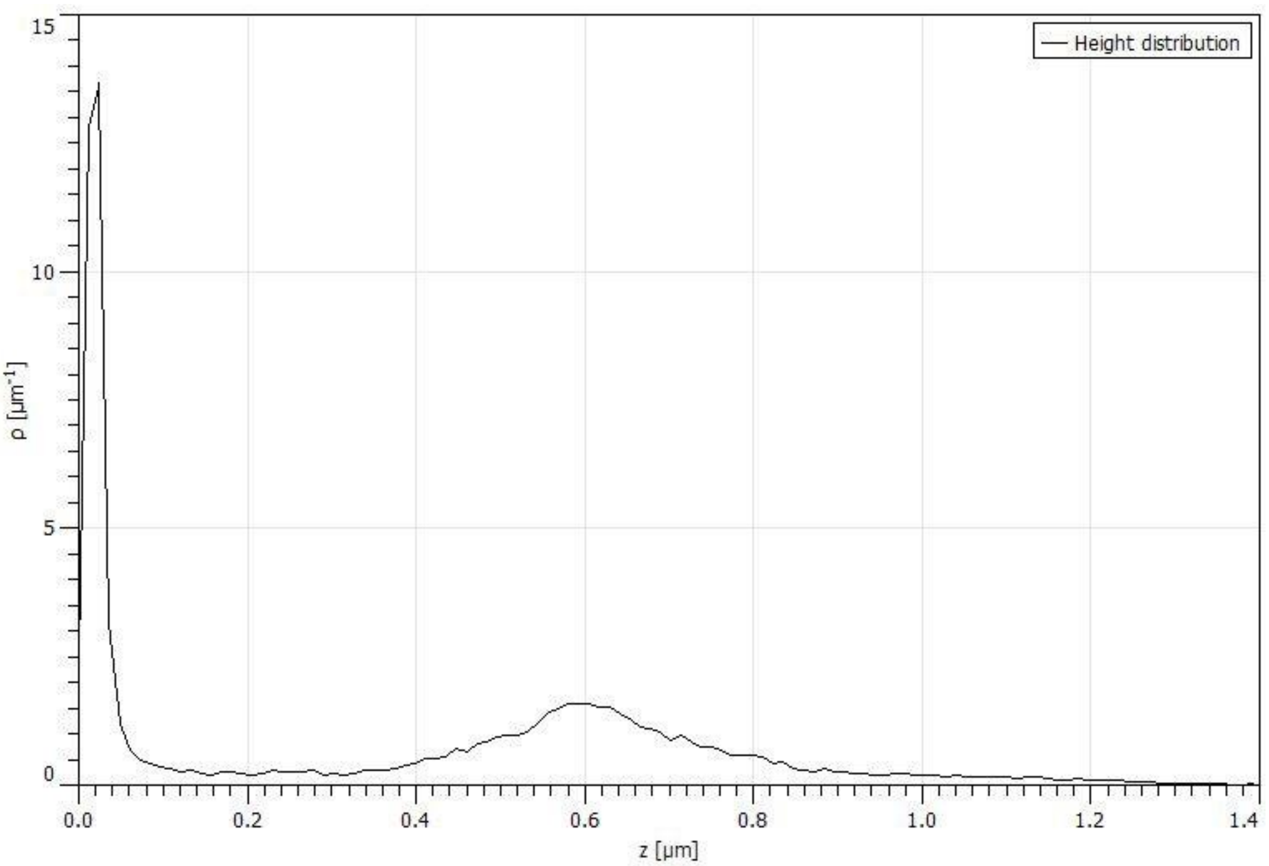

Figure S12: Representative example of histogram of height distribution for a printed structure after ligand exchange. The two visible peaks identify the substrate (sharp, 19 nm) and the main plateau of the structure (broader, 585 nm), resulting in a height measurement of 566 nm.

References

1 Hines, M. A.; Scholes, G. D. Colloidal PbS Nanocrystals with Size-Tunable Near-Infrared Emission: Observation of Post-Synthesis Self-Narrowing of the Particle Size Distribution. Adv. Mater. 2003, 15 566 (21), 1844−1849. https://doi.org/10.1002/adma.200305395

2 Kara, G.; Bolat, S.; Sharma, K.; Grotevent, M. J.; Dirin, D. N.; Bachmann, D.; Furrer, R.; Boesel, L. F.; Romanyuk, Y. E.; Rossi, R. M.; Kovalenko, M. V.; Calame, M.; Shorubalko, I. Conformal Integration of an Inkjet-Printed PbS QDs-Graphene IR Photodetector on a Polymer Optical Fiber. Adv. Mater. Technol. 2023, 8 (9), 483 2201922. https://doi.org/10.1002/admt.202201922

3 Sattler, K. D.; Anto, B. T.; Wong, L.-Y.; Rui-Qi, P.; Sivaramakrishnan, S.; Chua, L.-L.; Ho, P. K. H. *Handbook of Nanophysics. Functional Nanomaterials*; Taylor & Francis: Boca Raton, 2010. https://doi.org/10.1201/9781420075533.

4 Weidman, M. C.; Yager, K. G.; Tisdale, W. A. Interparticle Spacing and Structural Ordering in Superlattice Pbs Nanocrystal Solids Undergoing Ligand Exchange. *Chem. Mater.* **2015**, *27* (2), 474–482. https://doi.org/10.1021/cm503626s.
